# Regulation of proteolysis of the sigma factor RpoS by the Gac-Rsm signal transduction system in Azotobacter vinelandii

**DOI:** 10.1099/mic.0.001672

**Published:** 2026-02-20

**Authors:** Juliana Berenice Rojo-Rodríguez, Soledad Moreno, Guadalupe Espin

**Affiliations:** 1 Departamento de Microbiología Molecular, Instituto de Biotecnología, Universidad Nacional Autónoma de México, Cuernavaca, Morelos 62210, Mexico

**Keywords:** ClpP, Gac-Rsm, RpoS

## Abstract

In *Azotobacter vinelandii*, the sigma factor RpoS is maintained at low levels in exponentially growing cells due to degradation mediated by the chaperone–protease complex ClpXP, while high levels are observed in the stationary phase. This study showed that degradation of RpoS by ClpXP is under the control of the Gac-Rsm signal transduction system, in which GacA, the transcriptional activator of the two-component system GacS/GacA, activates transcription of the small RNAs RsmZ1 and RsmZ2. These RNAs bind to the translational repressor protein RsmA to counteract its repressor activity on its target mRNAs. We found that in stationary-phase cells, compared with the WT, a *gacA* mutant exhibited lower RpoS levels due to reduced stability, while levels of the *clpP* and *clpX* mRNAs were higher. Furthermore, inactivation of the *clpP* or *clpX* genes in the *gacA* mutant restored the stability of RpoS, suggesting that the observed RpoS instability is due to degradation by ClpXP. We also showed that inactivation of *rsmA* in either the WT or the *gacA* mutant resulted in RpoS levels higher than in the WT in both stationary and exponential phases, while *clpP* and *clpX* transcript levels were significantly reduced. Taken together, these data reveal that in *A. vinelandii,* the GacA-Rsm system controls RpoS stability through RsmA, which acts as a positive regulator of ClpXP expression.

## Introduction

*Azotobacter vinelandii* is a soil bacterium belonging to the *Pseudomonadaceae* family that produces the biodegradable thermoplastic polyhydroxybutyrate (PHB) during the stationary phase. In this bacterium, transcription of the *phbBAC* operon, which encodes the enzymes responsible for the synthesis of this polymer, is dependent on the stationary sigma factor RpoS [1,2]. In most Gram-negative bacteria, RpoS controls the general stress response [3]. Accordingly, RpoS protein levels increase during the stationary phase, which is a condition of nutrient limitation [4,6].

The regulatory mechanisms that control RpoS expression have been extensively studied in *Escherichia coli*, where regulation occurs at multiple levels, including transcription, translation and proteolytic degradation [7,10]. A proteolytic process mediated by the ClpXP ATP-dependent protease complex occurs in several Gram-negative bacteria [11,12], and in *E. coli* involves several proteins, including the RssB response regulator, which acts as an adaptor protein that binds RpoS and presents it to the ClpXP protease [13].

In *A. vinelandii,* as in other bacteria, degradation of RpoS by the ClpXP complex occurs during the exponential growth phase [14]. An operon encoding a RssB-like protein and RssC, a protein annotated as an anti-antisigma factor, was identified in species of the *Pseudomonadaceae* family [15]. In *A. vinelandii* and *Pseudomonas aeruginosa,* inactivation of *rssB* and/or *rssC* increases the levels of RpoS during exponential growth and affects RpoS-dependent phenotypes, such as PHB in *A. vinelandii* and pyocyanin in *P. aeruginosa* [15]. Moreover, an *in vivo* interaction between RssB and RpoS was observed only in the presence of RssC. Thus, it was proposed that both RssB and RssC are necessary for RpoS degradation by ClpXP during the exponential phase of growth in bacteria belonging to the *Pseudomonadaceae* family [15].

RpoS degradation in *A. vinelandii* was also shown to be under the control of the nitrogen-related phosphotransferase system (PTS^Ntr^) [14], a global regulatory system found in Gram-negative bacteria, composed of the EI^Ntr^, Npr and EIIA^Ntr^ proteins, encoded by the *ptsP*, *ptsO* and *ptsN* genes, respectively [16,18]. These proteins participate in a phosphorylation cascade starting from phosphoenolpyruvate, with EIIA^Ntr^ being the final acceptor [19]. In *A. vinelandii*, degradation of RpoS by the ClpAP chaperone–protease complex occurs during the stationary phase in mutants that carry an unphosphorylated EIIA^Ntr^ [14].

In species of the *Pseudomonas* genus, the Gac-Rsm signal transduction system, a global regulator of gene expression at the translational level, controls a wide variety of phenotypic traits [20,21]. The effector of this system is RsmA, an RNA-binding protein that interacts with its mRNA targets to repress their translation [22]. RsmA activity is controlled by small non-coding RNAs named RsmZ and RsmY, which sequester RsmA to counteract its repressor function [23]. Although RsmA control of translation is mostly negative, it can also be positive and directly activate translation. In *P. aeruginosa,* RsmA activates translation of *phz2*, which seems to be carried out by preventing the formation of a secondary structure that sequesters the Shine-Dalgarno SD sequence[24].

Transcription of the *rsmZ* and *rsmY* genes is activated by GacA, a response regulator of the GacS/A two-component system [25,27]. In *A. vinelandii*, the Gac-Rsm system regulates the synthesis of alginate, PHB polymers and the phenolic lipids alkylresorcinols. In this bacterium, RsmA binds to the mRNAs encoding proteins involved in the synthesis of these compounds to repress their translation, while the RsmZ1 and RsmZ2 RNAs bind to RsmA to neutralize its activity [28,30].

In this study, we provide evidence showing that in a *gacA* mutant of *A. vinelandii* derived from strain UW136, RpoS stability during the stationary phase is reduced due to its degradation by ClpXP, but not by ClpAP. Additionally, transcript levels of *clpP* and *clpX* are significantly increased. Furthermore, inactivation of *rsmA* increased RpoS stability during exponential growth and significantly reduced the *clpP* and *clpX* transcript levels, indicating that free (unsequestered) RsmA contributes to the reduced RpoS stability and upregulation of ClpXP observed in the *gacA* mutant.

Taken together, our results reveal that the differences in RpoS stability observed between the exponential and stationary growth phases are controlled by the Gac-Rsm system via a mechanism involving regulation of ClpXP expression.

## Methods

### Strains, media and culture conditions

The *A. vinelandii* strain used in this study was UW136 [31]. Bacterial strains and plasmids used or constructed in this study are listed in Table 1. *E. coli* TOP10 was used for plasmid isolation and maintenance. Media and growth conditions were as follows: *A. vinelandii* was grown at 30 °C in Peptone Yeast (PY) medium or Burk’s nitrogen-free salts [32] supplemented with 20 g l^−1^ sucrose (BS). Antibiotics were used at the following concentrations (in μg ml^−1^) for *A. vinelandii* and *E. coli*, respectively: spectinomycin, 50 and 100; kanamycin, 30 and 3; tetracycline, 40 and 15; gentamicin, 1 and 10; apramycin, 25 (not used for *E. coli*).

**Table 1.** Strains and plasmids used in this study

Strain	Description	Reference
TOP10	*E. coli*	Thermo Fisher Scientific
UW136	*A. vinelandii* WT strain	[31]
JGWS	UW136 carrying an *rpoS::Sp* mutation	[1]
UW*rsmA*	UW136 derivative carrying an *rsmA*::Gm non-polar mutation	This study
UW*gacA*	UW136 derivative carrying a *gacA*::Km mutation	This study
Ah*gacA*	UW136 carrying a *gacA*::Gm mutation	[29]
AH*gacA/gacA^+^*	AH*gacA* carrying plasmid pJETgacA-Tc cointegrated into the chromosome	This study
UW*rsmA-gacA*	UW*rsmA* with a *gacA*::Km mutation	This study
LSW1	UW136 carrying a *ptsP:*:Tc mutation	[14]
LMW33	UW136 carrying a *clpA::*Km mutation	[14]
LMW35	UW136 carrying a *clpP::*Km mutation	[14]
LMW39	UW136 carrying a *clpX::*Km mutation	[14]
UW*gacA-clpA*	LMW33 with the *gacA*::Gm mutation	This study
UW*gacA-clpP*	LMW35 with the *gacA*::Gm mutation	This study
UW*gacA-clpX*	LMW39 with the *gacA*::Gm mutation	This study
UW*rsmA*/*rsmA*^+^	UW*rsmA* carrying plasmid pJETrsmA-Tc cointegrated into the chromosome	This study
UW*rsmA-gacA*/*rsmA*^+^	UW*rsmA-gacA* carrying plasmid pJETrsmA-Tc cointegrated into the chromosome	This study
UW*rpoS::gusA-P1*	UW136 carrying a transcriptional *rpoS::gusA* gene fusion transcribed from the *rpoS* promoter P1	[38]
UW*rpoS::gusA-P2*	UW136 carrying a transcriptional *rpoS::gusA* gene fusion transcribed from the *rpoS* promoter P2	[38]
UW*rpoS::gusA-P3*	UW136 carrying a transcriptional *rpoS::gusA* gene fusion transcribed from the *rpoS* promoter P3	[38]
UW*gacA-rpoS::gusA-P1*	UW*gacA* carrying a transcriptional *rpoS::gusA* gene fusion transcribed from the rpoS promoter P1	This study
UW*gacA-rpoS::gusA-P2*	UW*gacA* carrying a transcriptional *rpoS::gusA* gene fusion transcribed from the *rpoS* promoter P2	This study
UW*gacA-rpoS::gusA-P3*	UW*gacA* carrying a transcriptional *rpoS::gusA* gene fusion transcribed from the *rpoS* promoter P3	This study
SAWZ1	UW136 with *rsmZ1-gusA* transcriptional fusion	[29]
SAWZ2	UW136 with *rsmZ2-gusA* transcriptional fusion	[29]
SAWGZ1	AHgacA with *rsmZ1-gusA* transcriptional fusion	[29]
SAWGZ2	AHgacA with *rsmZ2-gusA* transcriptional fusion	[29]
AHgacA-rsmZ1/gacA^+^	SAWGZ1 carrying plasmid pJETgacA-Tc cointegrated into the chromosome	This study
AHgacA-rsmZ2/gacA^+^	SAWGZ2 carrying plasmid pJETgacA-Tc cointegrated into the chromosome	This study
**Plasmid**		
pJET1.2	pJET1.2 blunt cloning vector	Thermo Scientific
pBSL97	Plasmid with a Km cassette	[33]
pBSL98	Plasmid with a Gm cassette	[33]
pJET*rsmA*	pJET with *rsmA* gene	This study
pJET*rsmA*::Gm	pJET with *rsmA*::Gm mutation	This study
pJET*gacA*	pJET with *gacA* gene Tc	This study
pJET*gacA*::Km	pJET with *gacA*::Km mutation	This study
pJET*rsmA*-Tc	pJET*rsmA* with a Tc cassette	This study
pUMAP1rpoS::*gusA*	Plasmid carrying a transcriptional rpoS::*gusA* fusion transcribed for the *rpoS* P1 promoter	[38]
pUMAP2rpoS::*gusA*	Plasmid carrying a transcriptional rpoS::*gusA* fusion transcribed for the *rpoS* P2 promoter	[38]
pUMAP3rpoS::*gusA*	Plasmid carrying a transcriptional rpoS::*gusA* fusion transcribed for the *rpoS* P3 promoter	[38]
pHP45-Ω-Tc	Plasmid with a Tc cassette	[35]
pK18mob	Mobilizable plasmid with a Km^R^ marker	[36]
pkgacA	Pk18mob carrying the gacA gene Km	This study
pRK2013	Mobilizing plasmid, Tra+ Km ^R^	[37]

*E. coli* strains were grown on Luria–Bertani medium at 37 °C.

### Construction of mutant strains and plasmids

Oligonucleotides used in this study are listed in Supporting Information Table S1, available in the online Supplementary Material.

To construct the UW*rsmA* strains, we amplified an 868 bp fragment containing the *rsmA* gene using RsmAUP and RsmADown oligonucleotides. The amplified fragment was cloned into plasmid pJET1.2 (Thermo Scientific). The resulting plasmid, pJET*rsmA*, was subjected to reverse PCR using RsmAInvFw and RsmAInvRv primers to introduce a gentamycin-resistant cassette (Gm) from plasmid pBSL98 [33]. The resulting plasmid, pJET*rsmA*::Gm, was linearized by digestion with *Sca*I and used to transform the WT *A. vinelandii* strain UW136, leading to the isolation of the UW*rsmA* strain.

The UW*gacA* strain was constructed as follows: UPgacA628bp and DowngacA600bp oligonucleotides were used to amplify the *gacA* gene, which was subsequently cloned into the pJET1.2 vector, yielding plasmid pJET*gacA*. To introduce a kanamycin-resistant cassette (Km) from plasmid pBSL97 [33] into the *gacA* gene, reverse PCR was performed using Fw:gacAinv and Rv:gacAinv oligonucleotides, resulting in a 47 bp deletion in *gacA* and generation of pJET*gacA*::Km. This plasmid was used to transform strain UW136, yielding the UW*gacA* mutant. The presence of the *gacA*::Km mutation was confirmed by PCR. 

The double mutant strain UW*rsmA-gacA* was isolated by transforming UW*rsmA* with total DNA from strain UW*gacA*. Gm- and Km-resistant transformants with a PHB-positive phenotype (Supporting Information Fig. S1) were selected, and the presence of the *gacA*::Km mutation was confirmed by PCR.

To construct UW*clpA-gacA*, UW*clpP-gacA* and UW*clpX-gacA* double mutant strains*,* LMW33*,* LMW35 and LMW39 strains were transformed with total DNA extracted from AH*gacA,* which carries a *gacA*::Gm mutation. Gm- and Km-resistant transformants were isolated and validated via PCR to confirm the *gacA::*Gm mutation.

Transformation of *A. vinelandii* strains was carried out as described by Page and Sadoff [34].

For the construction of *rsmA*-complemented strains, a DNA fragment containing the *rsmA* gene with its native promoter was amplified using primers RsmAUP/RsmADown, respectively. This fragment was cloned into the pJET1.2 vector, generating plasmid pJETrsmA. A tetracycline-resistant cassette (Tc) from plasmid pHP45-Ω-Tc [35] was inserted into the *Sca*I site of pJET1.2, resulting in plasmid pJETrsmA-Tc.

Because this plasmid does not replicate in *A. vinelandii*, strains UW*rsmA* and UW*rsmA-gacA* were transformed with pJET*rsmA*-Tc. Tetracycline-resistant transformants, UW*rsmA/rsmA*^+^ and UW*rsmA-gacA/rsmA^+^,* were isolated, and PCR analysis confirmed integration by a single recombination event of pJET*rsmA*-Tc in the corresponding Tc-resistant transformants.

For the construction of *gacA*-complemented strains, a *Bgl*II DNA fragment containing the *gacA* gene with its native promoter from plasmid pJETgacA was obtained. This fragment was cloned into the *BamH*I site of the mobilizable Km-resistant plasmid pK18mob [36]. The resultant plasmid, pKgacA, unable to replicate in *A. vinelandii,* was transferred to strains AHgacA, SAWGZ1 and SAWGZ2 by triparental conjugations mediated by the helper plasmid pRK2013 plasmid [37]. Kanamycin- and gentamycin-resistant transformants showing a PHB^+^ phenotype (Fig. S1), AHgacA/gacA^+^, AHgacA-rsmZ1-gusA/gacA^+^ and AHgacA-rsmZ2-gusA/gacA^+^, were isolated and confirmed by PCR analysis for the presence of the WT *gacA* gene.

To construct UW*gacA* derivatives carrying *rpoS::gusA* transcriptional fusions, plasmids pUMAP1rpoS::*gusA,* pUMAP2rpoS::*gusA* and pUMAP3rpoS::*gusA* [38], linearized by *Sca*I digestion, were used to transform the UW*gacA* strain. This resulted in UW*gacA-rpoS::gusA*P1, UW*gacA-rpoS::gusA*P2 and UW*gacA-rpoS::gusA*P3 strains. Integration of *rpoS::gusA* gene fusions by double homologous recombination was confirmed by PCR.

### PCR and qRT-PCR

Total RNA extraction was performed using the TRIzol^™^ Max^™^ Bacterial RNA Isolation Kit (Thermo Scientific) according to the manufacturer’s protocol. To eliminate genomic DNA contamination, RNA was treated with DNase (DNA-free^TM^, Ambion), and RNA concentration was measured by absorbance at 260 nm. RNA purity was assessed using the 260/280 nm absorbance.

cDNA synthesis was performed using the RevertAid ^TM^ First Strand cDNA Synthesis Kit (Fermentas Inc.) and gene-specific primers. Primer sequences for cDNA synthesis and real-time PCR (RT-PCR) are listed in Table S1. The cDNA was used as a template for RT-PCR. Quantification of fluorescence was performed on a LightCycler 480 System (Roche Diagnostics) using SYBR Green dye.

To determine transcript levels of *rpoS, clpP*, *clpX* and *clpA,* the following primers were used: *rpoS*: qRT-rpoSUp/qRTrpoSDown; *clpP:* FWqPCRclpP/RVqPCRclpP; *clpX*: FWqPCR-clpX2/RVqPCR-clpX2; *clpA*: qRT-clpA Up/qRT-clpA Down.

### Western blot assays

RpoS protein was detected by Western blot using polyclonal RpoS-6His antiserum, as described previously [14]. Total protein was extracted from cultures grown in PY after 8 h (exponential phase) or 36 h (stationary phase). To assess *in vivo* RpoS stability, apramycin (25 µg ml^−1^) was added to cultures to block protein synthesis. Protein concentrations were determined using the Lowry method [39].

## Results

### The effect of a *gacA* mutation on RpoS expression

Previously, a *gacA* mutation in an *A. vinelandii* strain ATCC9046 (non-isogenic to UW136) was found to exert a negative effect on the level of *rpoS* transcript, assessed by Northern blot analysis [40]. We investigated whether the expression of RpoS in *A. vinelandii* strain UW136 is under the control of the Gac/Rsm system by analysing the effect of a *gacA* mutation on *rpoS* transcription and on RpoS protein levels. In UW136, *rpoS* transcription was previously shown to initiate from three promoters (P1, P2 and P3), with P1 accounting for ~95% of *rpoS* transcriptional activity [38].

**Fig. 1.**
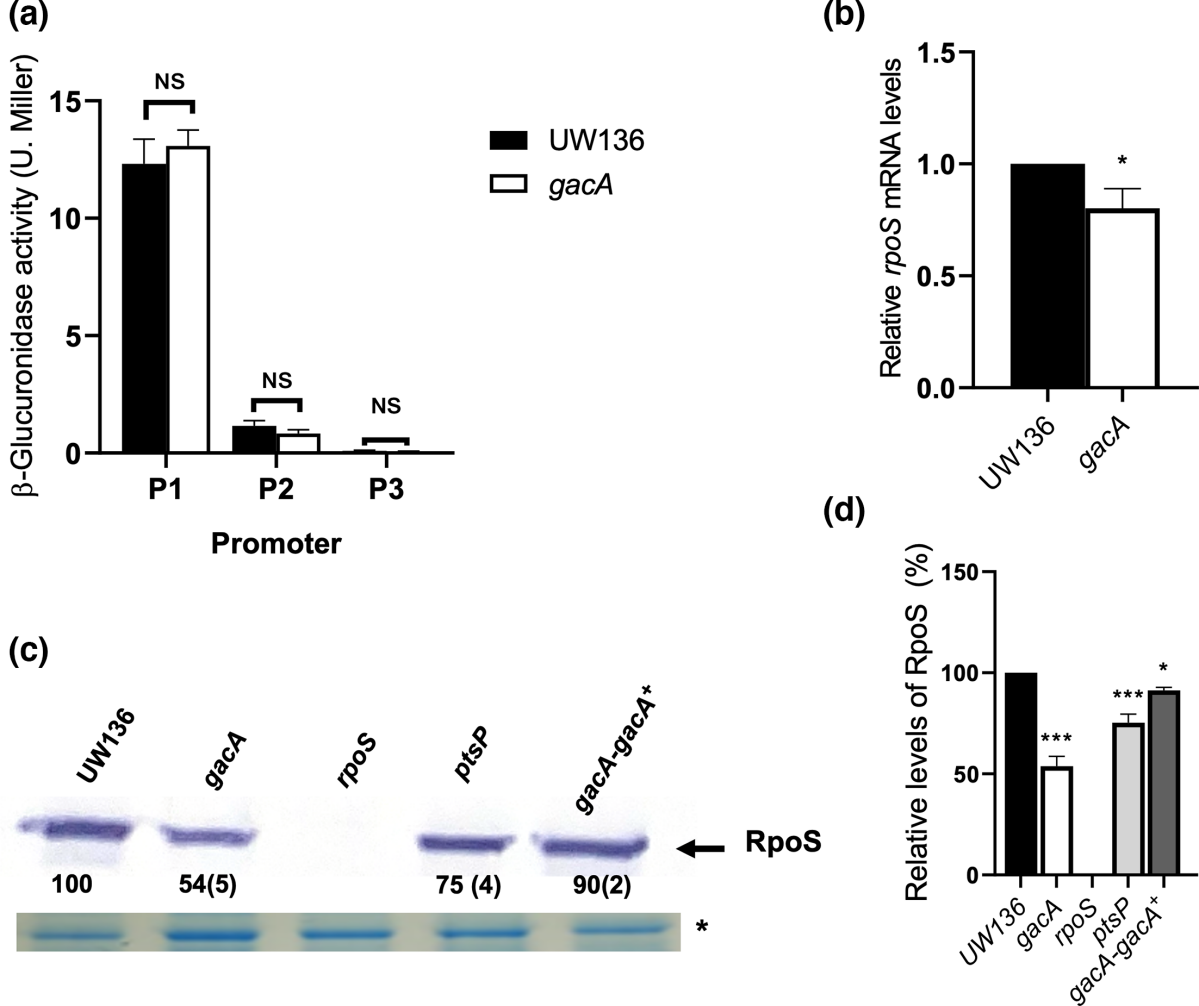
A mutation in *gacA* reduces RpoS protein level. (a) β-Glucuronidase activity in the UWgacA mutant and UW136 WT strains carrying *rpoS::gusA* transcriptional fusions to the P1, P2 and P3 *rpoS* promoters. (b) Relative expression of the *rpoS* gene, as determined by RT-qPCR in UWgacA and UW136 WT strains. (c) Detection of RpoS by Western blot in strains UW136, and in *gacA*, *rpoS, ptsP* and *gacA/*gacA^+^ mutants. Twenty micrograms of total protein was loaded per sample. RpoS levels in UW136 strain were considered 100%. Values represent the mean of three independent experiments, with sd shown in parentheses. The asterisk (*) indicates a protein loading control in a Coomassie-stained gel. For panels (a) and (b), data represent the mean of three independent experiments. Error bars indicate sd. Statistical significance was determined using an unpaired Student’s t-test (*P*<0.05). All experiments were performed with samples harvested after 36 h of growth in PY liquid medium at 30 °C. (**d**) Mean±sd of three independent biological replicates. Asterisks indicate statistically significant differences compared with WT [unpaired Student’s t-test for panels (a) and (b); one-way ANOVA with Dunnett’s post-hoc test for panel (d); *P*<0.05].

Strains UW136 and the UW*gacA* mutant carrying *rpoS::gusA* transcriptional fusions containing each of the three promoters were used to determine β-glucuronidase activity in stationary-phase cultures (36 h of growth). Unexpectedly, as shown in Fig. 1a, and in contrast to the ATCC9046 *gacA* mutant, under these conditions, the UW*gacA* mutant displayed *rpoS* transcription levels similar to those of the WT.

The level of *rpoS m*RNA was also measured by qRT-PCR in the *gacA* mutant and UW136 strains (Fig. 1b). A reduction of ~20% in *rpoS* transcript levels was observed in the *gacA* mutant compared with the WT.

The level of the RpoS protein was determined by Western blot analysis in stationary-phase cultures of UW136 and the *gacA* strains. We found that RpoS levels in the *gacA* mutant were reduced to ~54% of those observed in the parental WT UW136 strain; a reduction was also observed in strain LSW1, a *ptsP* mutant that carries an unphosphorylated EIIA^Ntr^ (Fig. 1c). To confirm that this effect was due to the absence of the GacA protein, we generated the complemented strain UW*gacA/gacA*^+^, in which the RpoS level was restored to 90% of the WT (Fig. 1c, d).

Taken together, these results suggest that the absence of GacA does not significantly affect *rpoS* transcription but causes a reduction in RpoS protein levels.

### RpoS instability in the *gacA* mutant is due to its degradation by ClpXP, not ClpAP

**Fig. 2.**
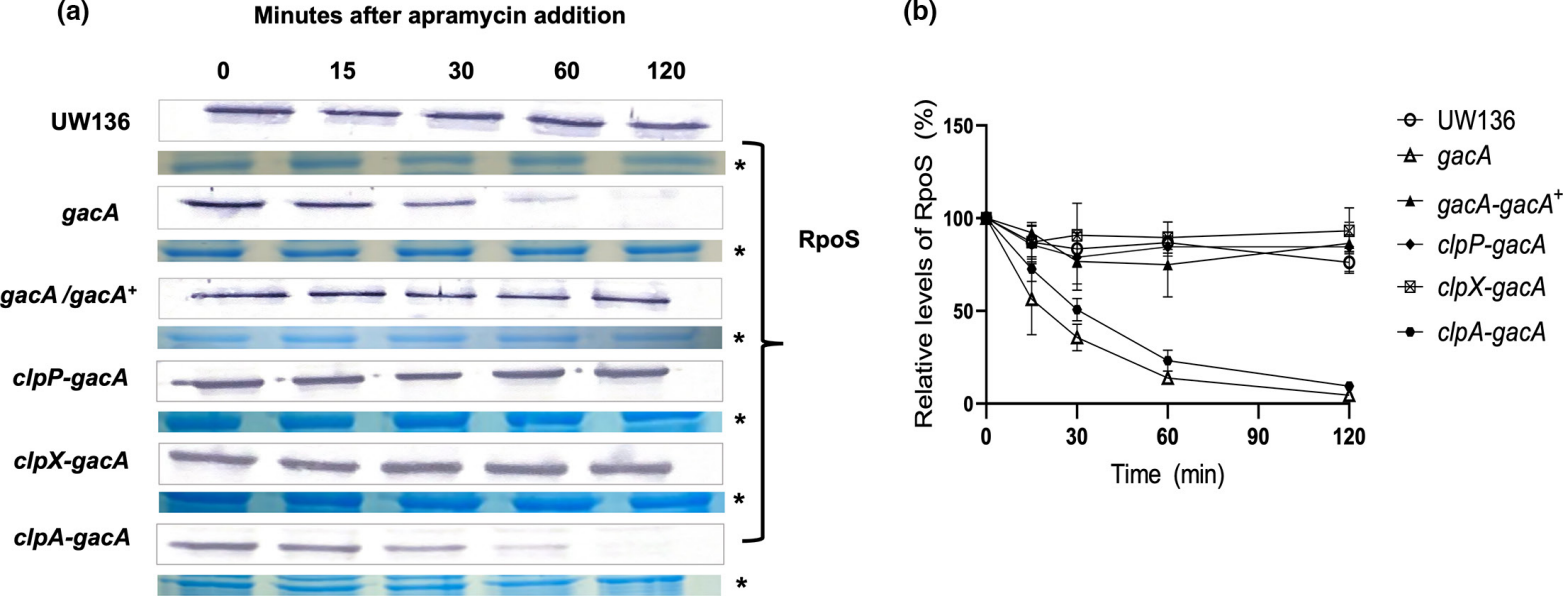
The reduction of RpoS level in the *gacA* mutant is caused by instability due to its degradation by ClpXP. (**a**) Determination of RpoS stability in the *gacA/gacA^+^* complemented strain and the *gacA* mutant and its derivatives carrying *clpA*, *clpX* and *clpP* mutations. RpoS levels were determined as described in Fig. 1. The asterisk (*) in panel (a) denotes a protein loading control in a Coomassie-stained gel. (b) Densitometric analysis of the experiment shown in panel (a). The mean of three independent experiments is presented. RpoS level at time 0 was considered 100%. Error bars indicate sd.

To determine whether the reduction in RpoS levels was due to protein instability, we assessed RpoS stability *in vivo* by adding apramycin to stationary-phase cultures to block protein synthesis. As shown in Fig. 2, RpoS levels remained stable in UW136 and UW*gacA/gacA*^+^ strains after apramycin addition, whereas in the *gacA* mutant, RpoS levels significantly declined after 60 min.

In *A. vinelandii* mutants derived from strain UW136 that carry unphosphorylated EIIA^Ntr^, such as *ptsP* mutants LSW1, RpoS stability is reduced due to its degradation by the ClpAP complex [14].

Additionally, it was previously shown that in a *gacA* mutant derived from *A. vinelandii* AEIV (a strain non-isogenic to UW136), EIIA^Ntr^ is found predominantly in an unphosphorylated state [41]. Therefore, we hypothesized that the reduction in RpoS stability observed in the *gacA* mutant derived from the UW136 strain could also be due to ClpAP-mediated degradation.

To test this, we constructed a *gacA-clpA* double mutant and evaluated RpoS stability *in vivo*. As shown in Fig. 2, inactivation of *clpA* in the *gacA* mutant did not restore RpoS stability, indicating that RpoS degradation in the *gacA* mutant is not mediated by ClpAP.

To investigate whether the ClpXP complex was responsible for the observed instability, we analysed RpoS stability in *gacA-clpP* and *gacA-clpX* double mutants. As shown in Fig. 2, inactivation of either *clpP* or *clpX* restored RpoS stability in the *gacA* mutant.

These results strongly suggest that ClpXP is responsible for RpoS degradation in the absence of GacA.

### GacA regulates the expression of *clpP* and *clpX*

In *A. vinelandii*, as in other bacteria, RpoS proteolysis by ClpXP occurs predominantly during the exponential growth phase [14]. As shown above, the reduced stability of RpoS in stationary-phase *gacA* mutant appears to be caused by ClpXP-mediated degradation. This raised the question of whether ClpXP expression is regulated by GacA.

**Fig. 3.**
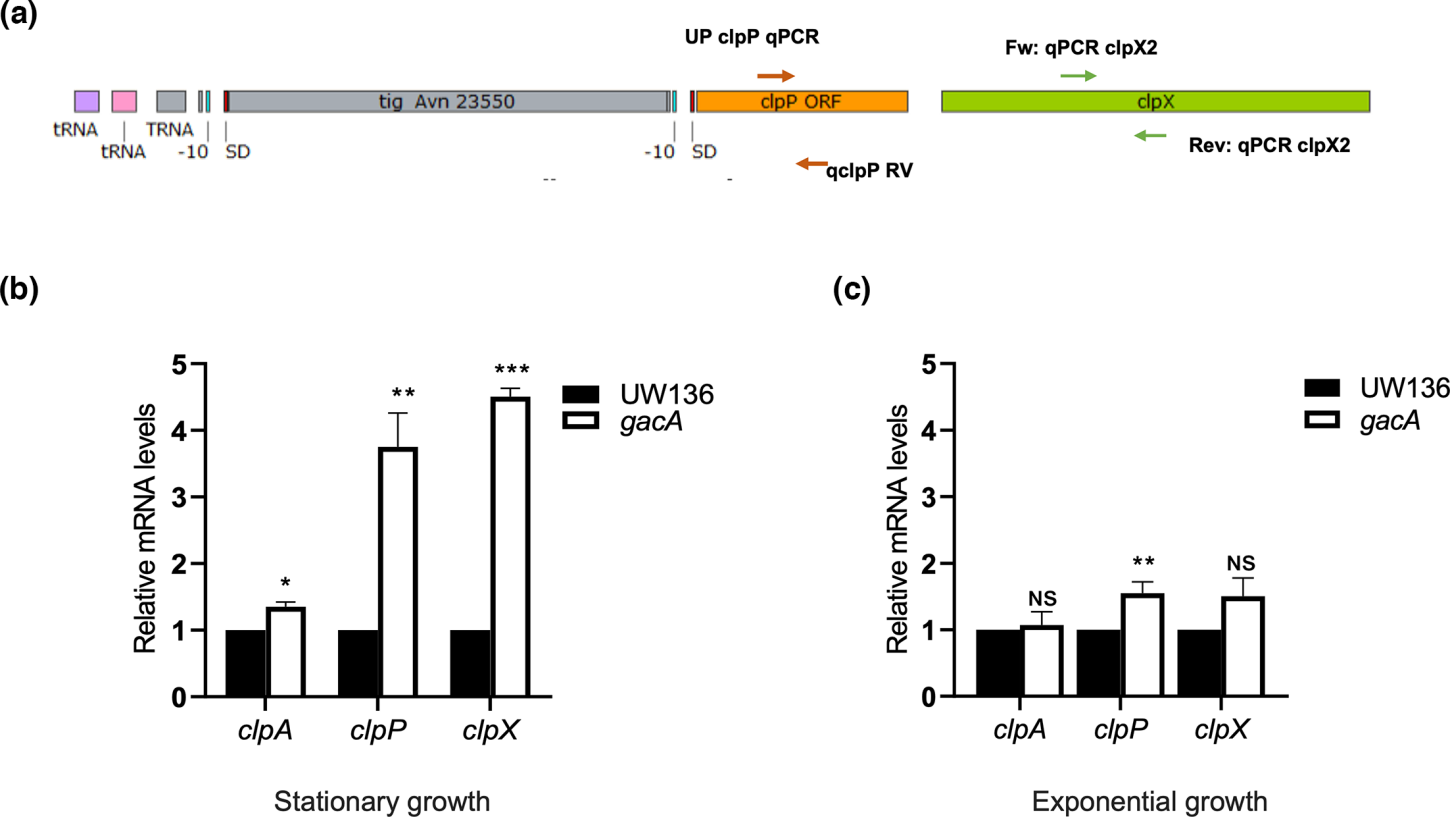
The *gacA* mutation increases *clpP* and *clpX* transcript levels during the stationary phase. (**a**) Schematic representation of the positions of oligonucleotides used in qRT-PCR to determine *clpP* and *clpX* transcript levels. (**b**) Relative expression of *clpP* and *clpX* genes measured by RT-qPCR in the *gacA* mutant during the stationary growth phase (36 h). (**c**) Relative expression of *clpA*, *clpP* and *clpX* genes measured by RT-qPCR in the *gacA* mutant during the exponential growth phase (8 h). Cells were grown in PY liquid medium at 30 °C. The mean of three independent experiments is presented. Error bars indicate sd. Asterisks indicate statistical significance (unpaired Student’s t-test) when comparing each transcript in the *gacA* mutant versus the WT strain (*P*<0.05).

We evaluated *clpA*, *clpX* and *clpP* transcript levels by RT-qPCR in WT UW136 and the *gacA* mutant. A schematic representation of the genetic context of *clpP* and *clpX* and the oligonucleotides used is shown in Fig. 3a. As shown in Fig. 3b, a ~fourfold increase in *clpP* and *clpX* transcript levels was observed in stationary-phase *gacA* mutant cultures compared with the WT. In contrast, *clpA* transcript level was only slightly elevated.

Transcript levels were also measured in exponential-phase cultures. A moderate (~50%) increase in *clpP* expression was observed in the *gacA* mutant compared with the WT (Fig. 3c). These results indicate that the *gacA* mutation strongly upregulates ClpXP expression during stationary phase but has only a modest effect during exponential growth. As expected, ClpA expression was not significantly affected.

### Effect of an *rsmA* mutation on *clpX* and *clpP* mRNA levels

**Fig. 4.**
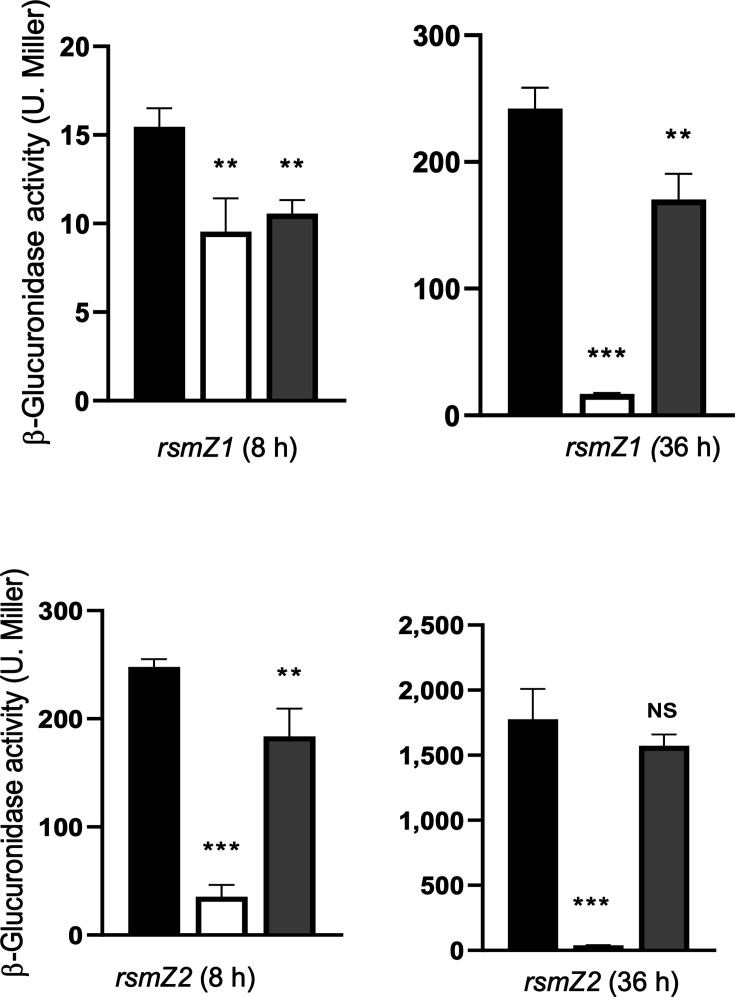
Transcription of the non-coding RNAs RsmZ1 and RsmZ2 is activated during the exponential phase and is dependent on GacA. β-Glucuronidase activity in strains UW136 (black bars) and AHgacA (white bars), carrying *rsmZ1-gusA* and *rsmZ2-gusA* transcriptional fusions, and complemented derivatives *gacA-rsmZ1::gusA*/gacA^+^ and *gacA-rsmZ2:gusA/gacA*^+^ (grey bars). β-Glucuronidase activity was determined in exponential (8 h) and stationary (36 h) phase cultures. Values represent the mean of three independent experiments; error bars, sd.

The translational regulatory activity of RsmA is controlled through its interaction with the small non-coding RNAs RsmZ, which counteract its function. In *A. vinelandii*, transcription of the RsmZ1 and RsmZ2 RNAs, as determined using *rsmZ-gusA* transcriptional fusions, occurs during stationary phase and is dependent on GacA [29] (Fig. 4). To confirm the positive regulation of *rsmZ* transcription by GacA, we constructed the complemented strains AHgacA-rsmZ1-gusA/gacA^+^ and AHgacA-rsmZ2-gusA/gacA^+^. As expected, transcription of *rsmZ1* and *rsmZ2* during the stationary phase was restored to WT levels in both strains (Fig. 4).

Accordingly, in exponential-phase cultures of the WT, RsmA is expected to be active and able to bind its target mRNAs. In contrast, during the stationary phase, RsmA becomes inactive due to sequestration by RsmZ1 and RsmZ2. In the *gacA* mutant, however, transcription of these RNAs is significantly reduced during stationary phase (Fig. 4), allowing RsmA to remain active (unsequestered) throughout both growth phases. Consequently, and consistent with the positive effect of the *gacA* mutation on *clpX* and *clpP* transcript levels described above, inactivation of *rsmA* was expected to affect the expression of these genes.

**Fig. 5.**
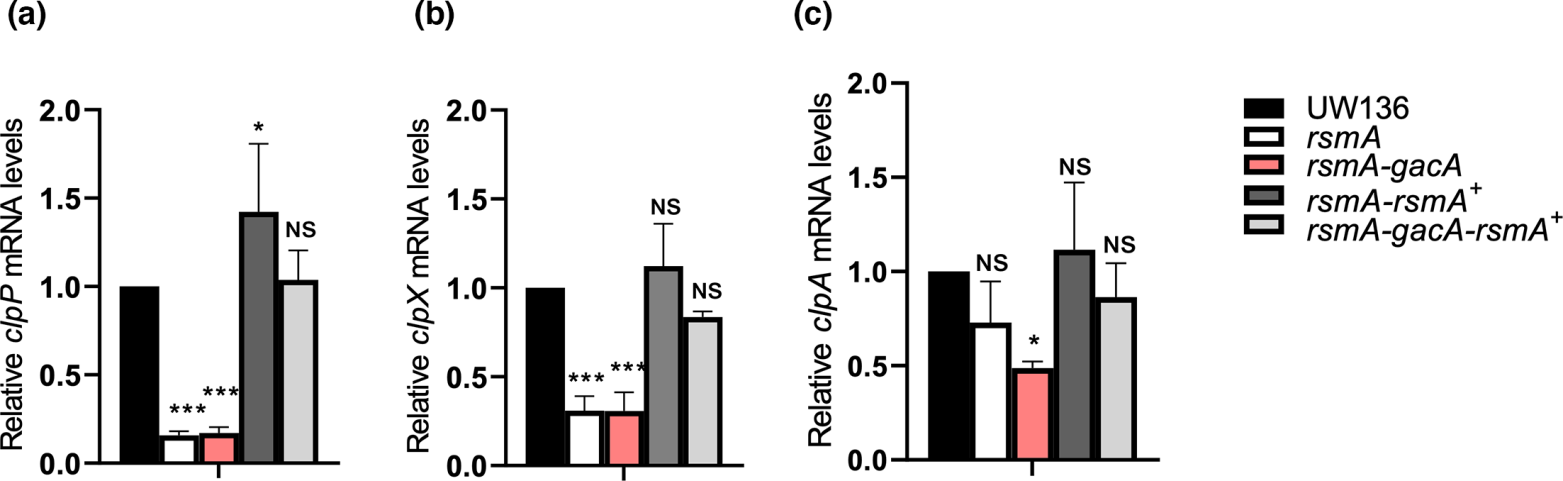
Expression of *clpP* and *clpX* is downregulated in the absence of RsmA. Transcript levels of *clpP* (**a**), *clpX* (**b**) and *clpA* (**c**), measured by RT-qPCR in *rsmA* and *rsmA-gacA* mutants, and in complemented strains *rsmA-rsmA^+^*and *rsmA-gacA-rsmA^+^*. All experiments were performed using samples collected after 36 h of growth in PY liquid medium at 30 °C. Data represent the mean of three independent experiments. Error bars indicate sd. Statistical significance was determined using an unpaired Student’s t-test (*P*<0.05).

As shown in Fig. 5, inactivation of *rsmA* caused a strong reduction in *clpP* and *clpX* transcript levels during stationary phase. This effect is opposite to that observed in the *gacA* mutant, in which RsmA remains in its active, unsequestered form. We also constructed an *rsmA-gacA* double mutant. As expected, in this strain, *clpP* and *clpX* transcript levels were similar to those observed in the *rsmA* single mutant (Fig. 5). To confirm that the reduction in *clpP* and *clpX* transcripts was due to the absence of RsmA, we generated the complemented strains UW*rsmA/rsmA*^+^ and UW*gacA-rsmA/rsmA*^+^. In both cases, *clpP* and *clpX* transcript levels were restored to WT levels, or even higher in the case of *clpP* in the UW*rsmA/rsmA*^+^ strain (Fig. 5).

Together, these findings indicate that, in the absence of RsmA, *clpP* and *clpX* expression is significantly downregulated, supporting the conclusion that RsmA acts as a positive regulator of ClpXP expression.

### Inactivation of *rsmA* restores RpoS expression in the *gacA* mutant

As shown above, ClpXP expression is downregulated in strains carrying an *rsmA* mutation. Therefore, we hypothesized that RpoS levels would be positively affected in these strains.

**Fig. 6.**
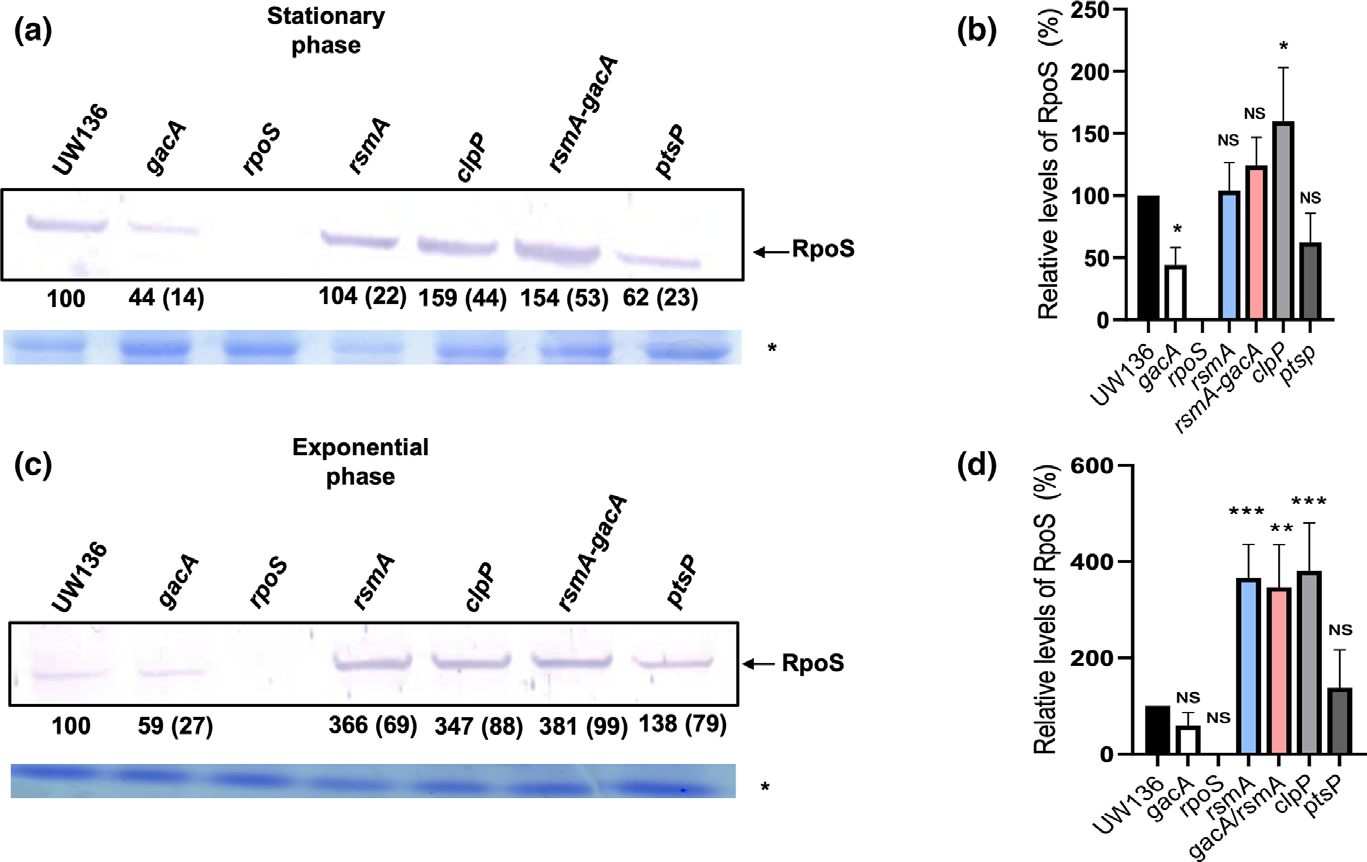
Inactivation of *rsmA* increases RpoS levels during the exponential phase and restores RpoS expression in the *gacA* mutant. Detection of RpoS by Western blot in strains UW136, *gacA*, *rpoS*, *rsmA, clpP, rsmA-gacA* and *ptsP* in (a) stationary phase (36 h) and (**c**) exponential phase (8 h) was carried out as described in Fig. 1c. Asterisks (*) in panels (a) and (c) indicate loading controls from Coomassie-stained gels. (**b and d**) Densitometric quantification of RpoS protein levels from panels (a) and (c), respectively. Data represent the mean±sd of three independent experiments. Asterisks in panels (c) and (d) indicate statistically significant differences compared with WT (one-way ANOVA with Dunnett’s post-hoc test; *P*<0.05).

We measured RpoS levels in stationary-phase cultures of *rsmA* and *rsmA-gacA* strains, where ClpXP-mediated proteolysis reduces RpoS levels in WT cells. Consistent with our hypothesis, in both *rsmA* and *rsmA-gacA* mutants, RpoS levels increased to values similar to those observed in the *clpP* mutant (Fig. 6 A and B).

We also analysed RpoS levels in exponential-phase cultures, as shown in Fig. 6 C and D.

RpoS levels in the *rsmA-gacA* double mutant were even higher than in the WT, while in the *rsmA* single mutant, they were similar to those of the WT.

These findings further support the conclusion that GacA-dependent regulation of RpoS proteolysis involves RsmA as a positive effector of *clpXP* expression.

## Discussion

The degradation of RpoS is tightly regulated at different stages of bacterial growth. In *E. coli*, during the exponential phase, the rapid degradation of RpoS by the ClpXP complex is mediated by the response regulator RssB [42], whereas in *A. vinelandii* and *P. aeruginosa*, both RssB and RssC participate in this process [15]. In contrast, the stationary-phase accumulation of RpoS mainly results from increased protein stability [4,43, 44]. However, the mechanism responsible for RpoS stabilization during this phase remains poorly understood.

In the present study, we describe a previously uncharacterized regulatory mechanism for the control of RpoS stability in *A. vinelandii*, mediated by the post-transcriptional regulatory system Gac-Rsm. We show that in a *gacA* mutant derived from UW136, RpoS protein levels decreased during the stationary phase. Although a negative effect of a *gacA* mutation on *rpoS* transcript levels, as determined by Southern blot analysis, was previously reported in *A. vinelandii* strain ATCC9046 [40], no significant effect on *rpoS* transcription was observed in strain UW136. Therefore, the reduction in RpoS levels in the UW136 *gacA* mutant is not due to transcriptional downregulation, but rather to decreased RpoS protein stability. The discrepancy in the *rpoS* transcript levels between ATCC9046 and UW136 *gacA* mutants may be explained by the fact that these strains are not isogenic.

In previous studies, we reported that the global regulatory PTS^Ntr^ system, composed of EI^Ntr^, Npr and EIIA^Ntr^ proteins and conserved in Gram-negative bacteria, controls RpoS stability. When unphosphorylated, EIIA^Ntr^ promotes ClpAP-dependent degradation of RpoS during the stationary phase [14]. Since in a *gacA* mutant derived from AEIV (a strain non-isogenic to UW136), EIIA^Ntr^ is predominantly unphosphorylated [41], we initially hypothesized that the reduced RpoS stability observed in the UW136 *gacA* mutant could result from ClpAP-mediated proteolysis. However, inactivation of *clpA* in the *gacA* mutant did not restore RpoS stability (Fig. 2), indicating that this pathway is not responsible. In contrast, inactivation of *clpP* or *clpX* genes in the *gacA* mutant restored RpoS stability (Fig. 2), implying that the instability of RpoS in the *gacA* mutant was due to ClpXP-dependent proteolysis rather than ClpAP activity.

In *Pseudomonas* species phylogenetically closely related to *A. vinelandii* [45], the post-transcriptional Gac-Rsm regulatory system controls diverse cellular processes. The response regulator GacA activates transcription of one or more small non-coding RNAs termed RsmZ, RsmY and RsmX. These RNAs sequester RsmA, a translational regulatory protein, thereby counteracting its repressor activity on target mRNAs [23,46,49].

In *A. vinelandii*, the Gac-Rsm system regulates the synthesis of PHB, alginate and alkylresorcinols by controlling translation of *algD, phbR* and *arpR* mRNAs through RsmA binding [28,30]. Expression of eight small RNAs (RsmZ1–7 and RsmY) is low during the exponential phase but strongly induced during the stationary phase in the WT UW136 strain, whereas this induction is absent in a *gacA* mutant. In addition, RsmA was shown to bind RsmZ1 and RsmZ2 [29]. Consequently, in the *gacA* mutant, RsmA remains active (unsequestered) in both growth phases, while in the WT, it is active only during the exponential phase.

Our results show that inactivation of *rsmA* increased RpoS levels during the exponential phase by ~3.5-fold, an effect comparable to that observed in a *clpP* mutant (Fig. 6). These findings indicate that RsmA promotes RpoS degradation by increasing *clpP* and *clpX* expression (Fig. 6).

Consistent with this interpretation, *clpP* and *clpX* transcript levels were elevated in the *gacA* mutant, where RsmA remains unsequestered and active, but were reduced in the *rsmA* mutant, correlating with the observed RpoS stability patterns (Figs1c, 5  6b). Although growth phase-dependent regulation of *clpP* and *clpX* transcription has been reported in other bacteria [50,51], regulation of *clpP* and *clpX* by the Gac-Rsm system has not previously been reported.

While RsmA in *A. vinelandii* generally functions as a translational repressor [28,30], the reduction of *clpP* and *clpX* transcripts in the *rsmA* mutant suggests a positive regulatory role on these genes at the level of transcription rather than translation. If this is the case, RsmA may regulate *clpP* and *clpX* expression indirectly, for example, by inhibiting the translation of a negative regulator. The possibility that RsmA might directly bind *clpP* and *clpX* transcripts to promote their translation, as reported for the *E. coli flhDC* or *P. aeruginosa phz2* transcripts [24,52, 53], is not ruled out. However, analysis of the Shine-Dalgarno regions and upstream *clpP* and *clpX* regions revealed no canonical RsmA-binding motifs corresponding to the SELEX-derived consensus sequence (CAMGGAYG) identified for *P. aeruginosa* RsmA targets [54].

**Fig. 7.**
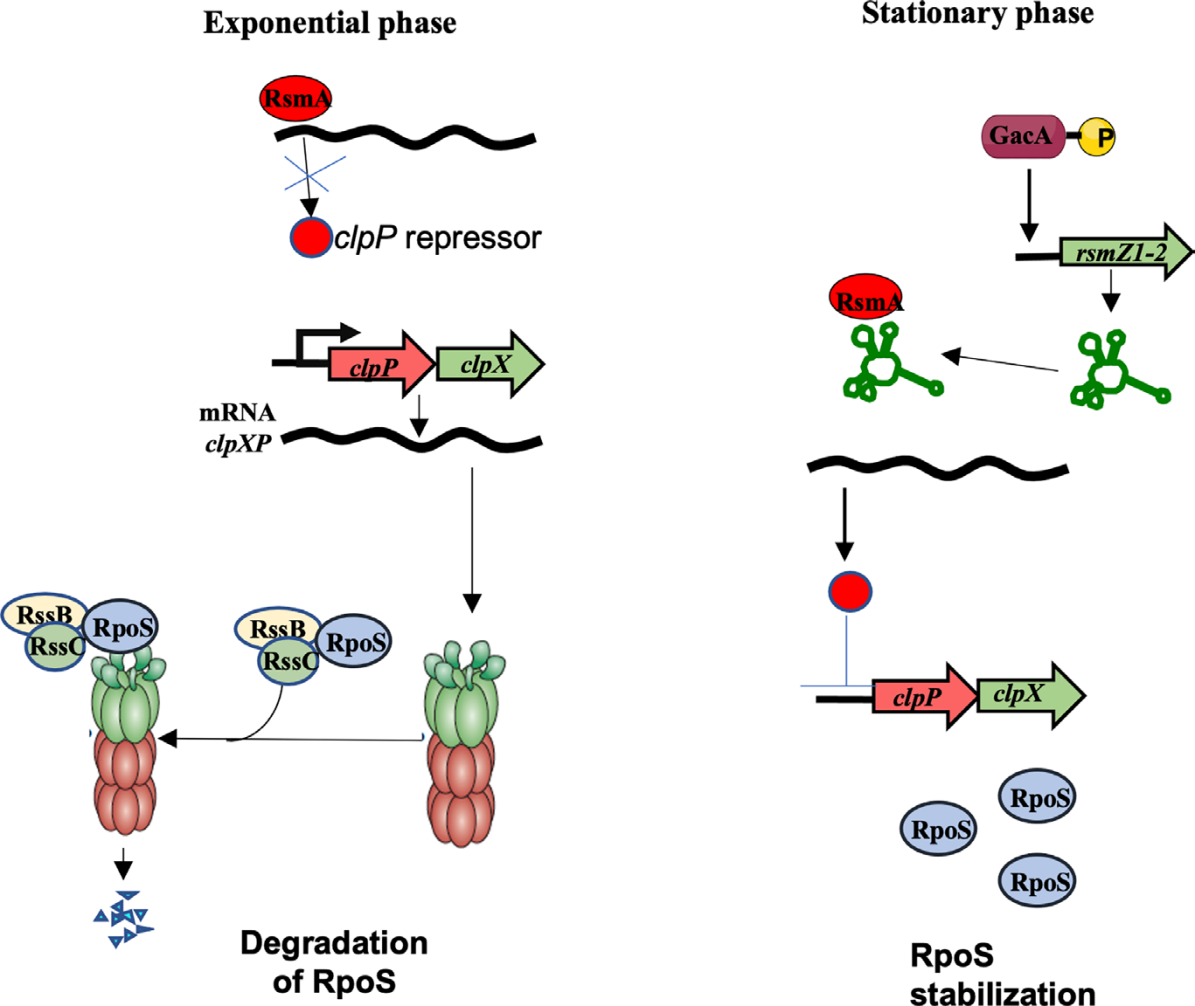
Proposed model for the regulation of RpoS levels during exponential and stationary phases. During the exponential growth phase, transcription of the *rsmZ* RNAs is significantly reduced. As a result, unsequestered RsmA protein might inhibit the translation of a putative repressor of *clpP* and *clpX* transcription, resulting in ClpXP-dependent degradation of RpoS. During the stationary phase, GacA is phosphorylated by GacS and activates transcription of the *rsmZ* genes. The accumulation of *rsmZ* RNAs results in sequestering RsmA, inhibiting its activity, thereby downregulating *clpXP* repression and allowing RpoS accumulation.

Taken together, our results support the model proposed in Fig. 7. During exponential growth, low RsmZ expression allows RsmA to remain unsequestered, enabling it to inhibit the expression of a putative negative regulator of ClpXP, leading to increased *clpP* and *clpX* transcription and, consequently, enhanced ClpXP-dependent degradation of RpoS. In the stationary phase, GacA induces *rsmZ* transcription, resulting in RsmA sequestration, repression of *clpP* and *clpX* and stabilization of RpoS. This regulatory strategy is consistent with the observation that transcription of *A. vinelandii* genes involved in alginate, PHB and alkylresorcinols synthesis initiates from RpoS-dependent promoters. Thus, in addition to post-transcriptional regulation of these genes by the Gac-Rsm system, this pathway indirectly promotes their optimal transcription by stabilizing RpoS.

Finally, an interplay between Gac-Rsm and RpoS regulons has also been reported in *Pseudomonas* species. However, in these bacteria, Gac-Rsm typically regulates RpoS at the transcriptional or translational level, rather than by controlling proteolysis, as shown in *Pseudomonas fluorescens*, *Pseudomonas chlororaphis*, *Pseudomonas putida* and *P. aeruginosa* [55,59].

## Supplementary material

10.1099/mic.0.001672 Uncited Supplementary Material 1.

## Abbreviations

Gm: gentamycin-resistant cassette
Km: kanamycin-resistant cassette
NS: No Significanse
PHB: polyhydroxybutyrate
PTS^Ntr^: nitrogen-related phosphotransferase system
PY: Peptone Yeast
RT-PCR: real-time PCR
Tc: tetracycline-resistant cassette
