# Supplementary material for: Regulation of proteolysis of the sigma factor RpoS by the Gac-Rsm signal transduction system in Azotobacter vinelandii
Source: Microbiology (Reading). 2026 Feb 20;172(2):001672. doi: 10.1099/mic.0.001672 (PMC12923157; doi:10.1099/mic.0.001672)
Supplement: Uncited Supplementary Material 1. [file mic-172-01672-s001.pdf]

Table S1. Oligonucleotides used in this study

Name	Sequence 5'→3'	Reference
RsmADown	CGTGCGCATTATAGTGTCAT	This study
RsmAUP	CGCACGCGAAGTCATTGGC	This study
rsmAInvFw	CACCGCGAAGAAATCTACCAGC	This study
RsmAInvRv	GATGCGAACCTGATTACCTTTCAC	This study
qRT-rpoS Up	AGG ATG TCC TGG ACG ATG AG	33
qRT-rpoS Down	TCC AGC GCC CTA GTG TAG TC	33
UPgacA 628pb	CACGGTGAAATAGACGGCACC	This study
DowngacA 600pb	GCCCTTCGAGAAACATCACCG	This study
Fw:gacAinv	CGGGCATCGCTATATCAGCCC	This study
Rv:gacAinv	CTTGGTCAGGTAGCCAGCCG	This study
FWqPCR clpP	GGCGAACTTGATTGTCGCT	This study
RVqpcR clpP	GCTTGATCAACTGCATGGTG	This study
FWqPCRclpX2	GGTTATGTTGGCGAGGAT	This study
RVqPCRclpX2	GGATTTGCGCGAAATCTTGTC	This study
qRT-clpA Up	GCATGACTTGCAGGAGTTCA	This study
qRT-clpA Down	CACTTCCCGCTTACCAGAAC	This study

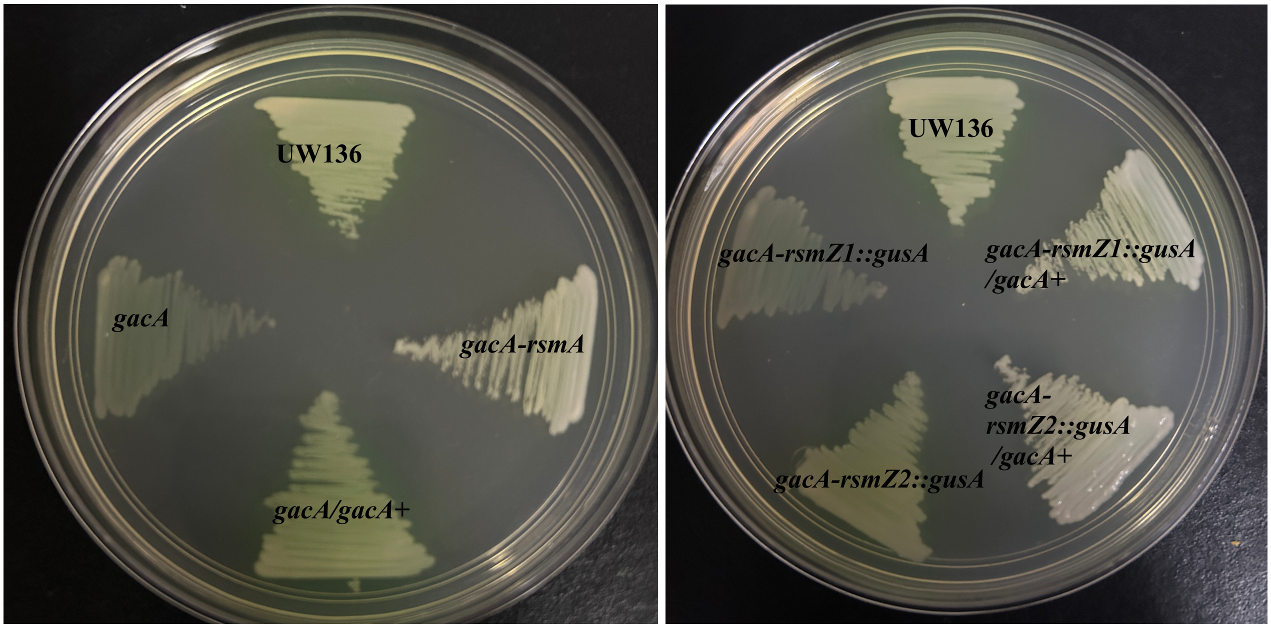

Figure S1. Polyhydroxybutyrate phenotype of *gacA-rsmA* and *gacA* complemeted mutans.

PHB synthesis phenotype of strains grown in Peptone-Yeast medium 2% sucrose (PY) for 48 hours a 30° C.
A white color is compatble with PHB accumulation showed by wild type strain UW132, while a less white appearence means reduction or no PHB accumulation
